# Behavioural intervention in medication overuse headache: A concealed double‐blind randomized controlled trial

**DOI:** 10.1111/ene.15256

**Published:** 2022-02-10

**Authors:** Judith A. Pijpers, Dennis A. Kies, Erik W. van Zwet, Frits R. Rosendaal, Gisela M. Terwindt

**Affiliations:** ^1^ Department of Neurology Leiden University Medical Centre Leiden the Netherlands; ^2^ Department of Radiology Leiden University Medical Centre Leiden the Netherlands; ^3^ Department of Medical Statistics Leiden University Medical Centre Leiden the Netherlands; ^4^ Department of Clinical Epidemiology Leiden University Medical Centre Leiden the Netherlands

**Keywords:** behavioural intervention, chronic migraine, headache nurse, medication overuse headache, withdrawal therapy

## Abstract

**Background and purpose:**

Medication overuse headache is a prevalent disorder, with a strong biobehavioural component. Hence, behavioural interventions might effectuate reduction of the overused medication. We assessed in a double‐blind manner the efficacy of a behavioural intervention during medication withdrawal therapy.

**Methods:**

In this concealed, double‐blind, randomized controlled trial in medication overuse headache, conducted at the Leiden University Medical Centre, we compared the effect of maximal versus minimal behavioural intervention by a headache nurse during withdrawal therapy. Maximal intervention consisted of an intensive contact schedule, comprising education, motivational interviewing, and value‐based activity planning during 12 weeks of withdrawal therapy. Minimal intervention consisted of a short contact only. Patients were unaware of the existence of these treatment arms, as the trial was concealed in another trial investigating botulinum toxin A. Endpoints were successful withdrawal and monthly days of acute medication use after the withdrawal period.

**Results:**

We enrolled 179 patients (90 maximal, 89 minimal intervention). At Week 12, most patients achieved withdrawal in both groups (82/90 [93%] maximal intervention vs. 75/89 [86%] minimal intervention, odds ratio = 2.44, 95% confidence interval [CI] = 0.83–7.23, *p* = 0.107). At Week 24, patients in the maximal intervention group had fewer medication days (mean difference = −2.23, 95% CI = −3.76 to −0.70, *p* = 0.005). This difference receded over time. Change in monthly migraine days did not differ between groups (−6.75 vs. −6.22).

**Conclusions:**

This trial suggests modest benefit of behavioural intervention by a headache nurse during withdrawal therapy for medication overuse headache, to reduce acute medication use during and shortly after intervention, but extension seems warranted for a prolonged effect

## Graphical abstract

A double‐blind, randomized controlled trial performed by Pijpers et al. suggests that behavioural intervention reduces medication use in patients with medication overuse headache.

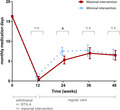

## INTRODUCTION

To reduce the burden of chronic disorders, many nonpharmacological interventions, such as behavioural therapy, lifestyle intervention, and mindfulness, are being studied and suggested to be effective [1, 2, 3]. Similarly, in headache disorders, psychological treatment seems beneficial, although recommendations on these therapies are hampered due to the quality of available research [3]. A major concern regarding research in this field is the risk of bias by awareness of the received treatment, as it is difficult to perform blinded trials due to the nature of the intervention [3]. As such, evidence is mainly based on observational or nonblinded randomized controlled trials. Therefore, it remains difficult to distinguish the specific effect of therapy itself from that of other factors, such as underlying expectations and receiving attention [4], which is especially important in trials on various disorders of the central nervous system [5].

Implementation of behavioural interventions might be particularly relevant in the care of headache patients with medication overuse headache (MOH). Medication overuse, the regular use of acute headache medication on at least 15 days per month in the case of simple analgesics or at least 10 days per month in the case of triptans, ergotamine, combination analgesics, opioids, or a combination of medication classes for >3 months [6], aggravates and maintains chronic headache [7, 8]. Epidemiological data suggest that overuse of analgesics and other pain medication is common, as approximately 1% of the general population suffers from MOH [9]. Medication overuse is a major risk factor for transformation from episodic migraine to chronic migraine (CM; i.e., headache on 15 or more days per month, of which at least 8 days fulfil migraine criteria) [6, 7, 10]. Withdrawal of the overused medication is an important step in medical care, with possibly added effect of preventive medication during the withdrawal process [7, 8, 11]. Overuse of pain medication has a strong biobehavioural component [12, 13, 14], and withdrawal therapy in itself requires significant adjustments in behaviour and lifestyle. Furthermore, a brief intervention in primary care reduced medication overuse in MOH patients. As such, the addition of a behavioural and educational intervention during withdrawal therapy is likely beneficial, but has mostly been studied in observational trials [15, 16]. An open‐label study failed to prove superior effects of an educational programme relative to standard treatment among MOH patients after withdrawal, but found that patients perceived increased efficacy in the use of their coping strategies to control pain [17, 18]. We report a concealed double‐blinded randomized trial to study the efficacy of a behavioural intervention during acute medication withdrawal, with and without botulinum toxin A (BTX‐A), in MOH patients with underlying migraine using a new study design that ensures blinding.

## METHODS

### Study design and patients

This was a concealed, randomized, double‐blind controlled clinical trial conducted at the Leiden University Medical Centre, as part of the Chronification and Reversibility of Migraine (CHARM) study [19] (trial register identifier: www.trialregister.nl, NTR3440). Patients aged 18–65 years, diagnosed with MOH and CM according to the International Classification of Headache Disorders, third edition criteria [6] were enrolled between December 2012 and February 2015. Main exclusion criteria were (i) other neurological disorders; (ii) other major comorbidity (chronic pain, psychiatric disorders, apart from depression and/or anxiety, cognitive, behavioural, or oncologic disorders); (iii) regular use of ergots, opioids, or barbiturates; and (iv) abuse of illicit drugs in the past 12 months.

### Procedures and intervention

**FIGURE 1:**
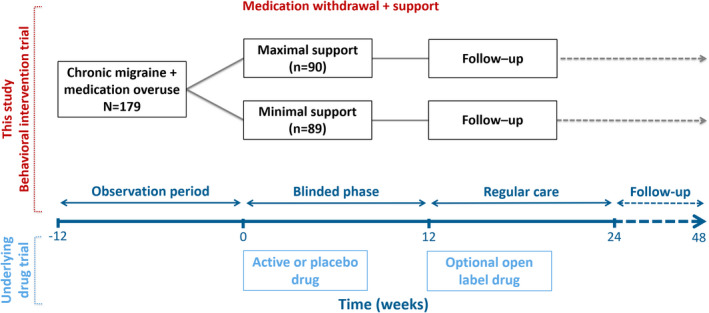
Study design. The blinded phase (Weeks 0–12) included medication withdrawal therapy plus maximal or minimal intervention, randomized by a centralized schedule using a design with blocks of four to eight patients, stratified for gender and treatment allocation in the drug trial. Hence, in both groups, half of the patients received active drug botulinum toxin A (BTX‐A; 31 injections, 155 units), and half of the patients received placebo drug (saline + low dose BTX‐A in the forehead region to ensure blinding; 24 injections with saline plus seven injections with BTA, 17.5 units) [19]. The drug was administered at the initiation of withdrawal. Regular care (Weeks 12–48) entailed advice to restrict use of acute medication (on ≤4–8 days per month) to prevent relapse into medication overuse, and, if necessary, initiation of prophylactic treatment. Patients who succeeded in withdrawing, but still suffered from chronic migraine, could receive open‐label drug (BTX‐A) as prophylactic treatment. Regular care typically comprises four to eight outpatient contacts per year by the treating physician [Colour figure can be viewed at wileyonlinelibrary.com]

Patients started with a 4‐week baseline assessment period, followed by the 12‐week withdrawal period. Medication withdrawal was implemented according to the national guidelines and other withdrawal studies [15, 20, 21, 22, 23], comprising abrupt cessation of any acute headache medication and no allowance for escape medication. In case of use of prophylactic drugs, these were tapered off. During this withdrawal period, patients were randomized to receive either maximal or minimal intervention by a headache nurse. In this study setting, a headache nurse is specifically educated for headache care with additional training on cognitive behavioural therapy and motivational interviewing, with at least some years of experience. Maximal intervention by a headache nurse consisted of a 30‐min consult immediately after the neurologist's interview, examination, and advice to withdraw, with at least three follow‐up telephonic contacts (every 2–4 weeks) during withdrawal. These consults were used to reiterate the withdrawal advice, educate patients on the risks of medication overuse and expected course of the withdrawal period, and increase intrinsic motivation to initiate medication withdrawal using motivational interviewing techniques. Furthermore, an individualized plan of approach was developed, acknowledging the influence of chronic migraine and withdrawal on professional and social life and enhancing acceptance. Alternative behavioural strategies to cope with the untreated pain were discussed, and a value‐based approach was used to establish activities during the withdrawal period. Minimal intervention consisted of a single consult of ≤15 min and no offer of follow‐up contacts by the nurse, mainly focusing on the reprise of the withdrawal advice and education on medication overuse. Patients of both treatment groups were provided with contact details to reach the hospital at any time if needed. Patients were unaware of the existence of these two treatment arms, because the study was concealed within a drug trial, studying the added effect of BTX‐A to acute withdrawal therapy in a randomized placebo controlled manner (see Figure 1 and reported in detail elsewhere [19]). Subsequent to the 12‐week withdrawal period, restricted use of acute medication was advised and prophylactic treatment was initiated if necessary. In both treatment groups, behavioural intervention by a headache nurse was not continued after the 12‐week period, but regular care was provided by the treating physician. Patients who continued to have CM after successful withdrawal were offered one treatment with open‐label BTX‐A. There were no differences between groups of patients treated with BTX‐A and placebo on any of the outcome measurements in both the double‐blind phase (after 12 weeks) or long‐term, open‐label phase (after 24, 36, and 48 weeks) [19].

Study follow‐up visits were planned after 12, 24, and 48 weeks. Patients kept 4‐week diaries with daily registration of headache characteristics and use of acute headache medication during the baseline assessment period and at Weeks 9–12, 21–24, 33–36, and 45–48.

### Randomization and masking of intervention

According to a centralized randomization schedule, patients were randomized 1:1 to receive either maximal or minimal behavioural intervention by a headache nurse, using blocks of four to eight patients, stratified for sex and the allocated treatment in the drug trial, ensuring that half of the patients in each group received BTX‐A. Patients were unaware of the existence of the two treatment arms, as this study was concealed within the drug trial studying BTX‐A, guaranteeing blinding of patients. Redundant both maximal and minimal behavioural intervention are interventions without any risk of harm, both fulfilling standard care for medication withdrawal, and patients were informed that the data of the CHARM study were to be analysed for a variety of research questions. Treating physicians and observers were blinded to treatment allocation and did not have access to the randomization schedule.

### Ethical statement

The study was performed in accordance with the Declaration of Helsinki Ethical Principles and Good Clinical Practices. The study design, including the concealment of this study and the corresponding informed consent, was approved by the local and national ethics committees (Medical Ethics Committee of the Leiden University Medical Centre and Central Committee on Research Involving Human Subjects, respectively).

### Outcome measures and analysis

The predefined outcome measures were successful withdrawal after 12 weeks and monthly days with use of acute headache medication after the withdrawal period. Successful withdrawal was defined as intake of acute medication on ≤2 days/month. Change in monthly days of acute medication use was estimated at timepoints 12, 24, 36, and 48 weeks. Because the intervention aims to enhance medication withdrawal and focuses on medication‐related behaviour, all outcomes of this study were related to medication use. A previous retrospective study by our group indicated that intervention by a headache nurse increases withdrawal adherence, but does not directly influence migraine headache frequency [15]. In line with this, the drug trial [19] and the current trial had different aims; the drug trial focusing on the effect of treatment on headache frequency, and the current trial focusing on the effect of intervention on medication use‐related outcomes. Although adjustment for the concurrent trials may technically not be necessary due to the randomization process, we chose to adjust for drug treatment allocation in all analyses, as we did correct for the behavioural intervention in the previous trial [19]. To provide a comprehensive overview, the monthly migraine days after withdrawal therapy and during follow‐up will be depicted in this study. For elaborate analysis on various outcome measures upon withdrawal therapy, we refer to the drug trial [19]. Descriptive data are reported as mean ± SD or number with proportion, and differences between groups are shown with 95% confidence intervals. Multivariate regression models were fit adjusting for age, gender, baseline medication days, drug treatment allocation, and depression and anxiety (based on the Hospital Anxiety and Depression Scale) [24]. For the repeated measures model, unstructured covariance matrixes were used. Analyses were (modified) intention‐to‐treat, including patients who provided at least one outcome measurement, performed in SPSS 23.0 (IBM).

## RESULTS

**FIGURE 2:**
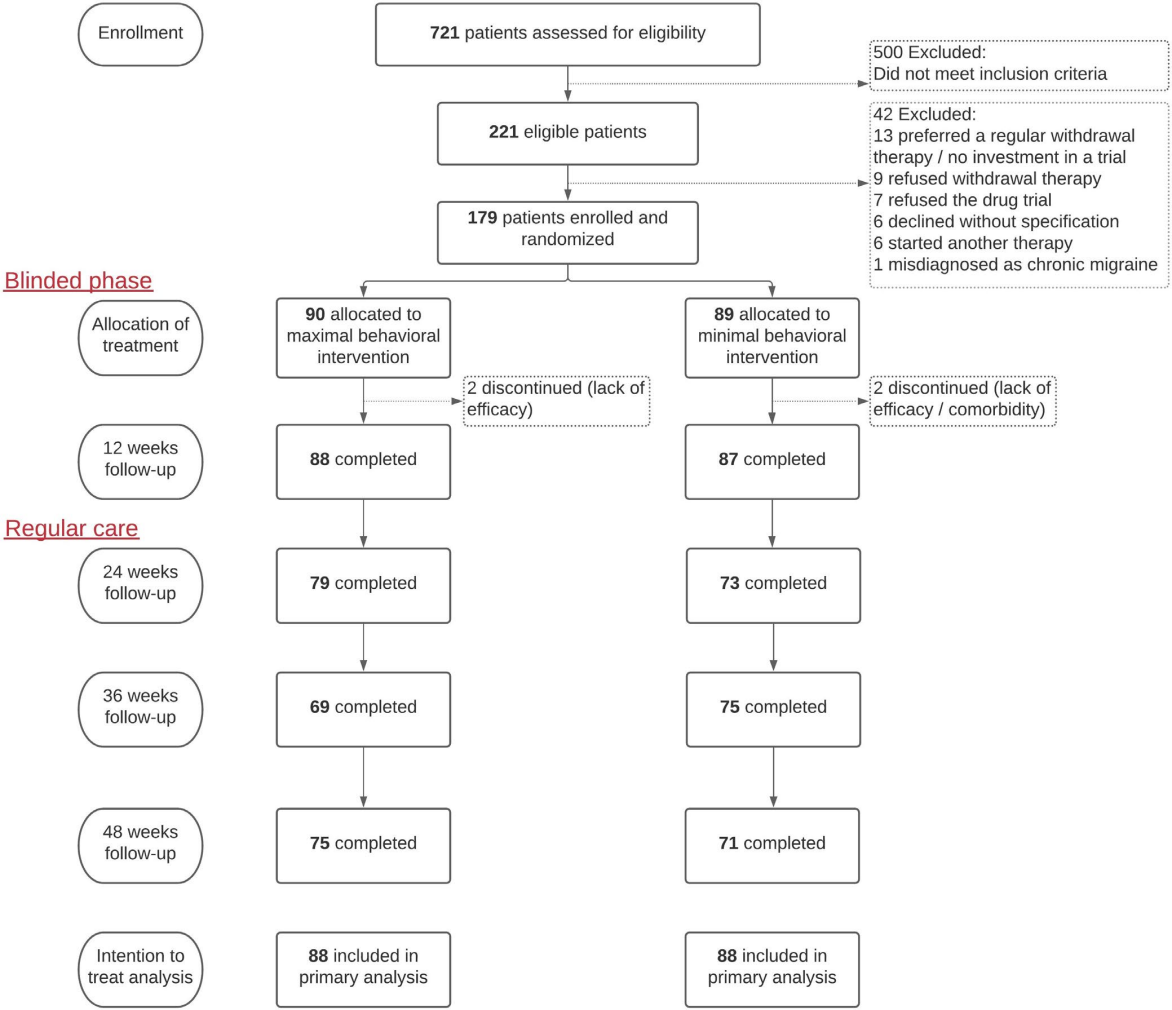
Flowchart [Colour figure can be viewed at wileyonlinelibrary.com]

Of 179 MOH patients, 90 were allocated to receive maximal intensive behavioural intervention and 89 to minimal intervention during the first 12 weeks of withdrawal therapy (Figure 2). Patients in the two treatment arms did not differ in sex, age, headache characteristics, medication use, and psychiatric comorbidity. Allocation to the two treatments arms within the drug trial was well‐balanced between the two groups, with an equal distribution of BTX‐A and placebo (Table 1). Also, the administration of open‐label BTX‐A was equally divided between groups (maximal intervention, *n* = 28; minimal intervention, *n* = 28). Follow‐up was complete for 98% (*n* = 175) after 12 weeks and 82% (*n* = 147) after 48 weeks, with similar numbers of dropouts in both groups. Most patients (88%) were accurately treated according to the protocol of the allocated treatment (maximal intervention, *n* = 82 [91%]; minimal intervention, *n* = 75 [84%]).

**TABLE 1:** Baseline demographic and clinical characteristics

Characteristic	Maximal intervention, *n* = 90	Minimal intervention, *n* = 89
Gender, female	67 (74.4%)	69 (77.5%)
Age, years	45.3 ± 10.9	45.1 ± 10.7
Monthly headache days	21.3 ± 4.6	21.5 ± 4.9
Monthly migraine days	15.3 ± 5.5	14.9 ± 5.5
Duration of migraine, years	27.3 ± 13.0	27.9 ± 12.9
HIT‐6 score^a^	65.3 ± 4.4	64.7 ± 4.1
Treatment within drug trial		
Botulinum toxin A	45 (50%)	45 (50.6%)
Placebo	45 (50%)	44 (49.4%)
Monthly days with acute headache medication	16.7 ± 5.6	16.2 ± 5.6
Type of overuse		
Triptans	18 (20.0%)	15 (16.9%)
Simple analgesics^b^	2 (2.2%)	5 (5.6%)
Combination of acute medication^c^	70 (77.8%)	69 (77.5%)
Prophylaxis^d^		
Current use	29 (32.2%)	36 (40.4%)
History of use^e^	84 (93.3%)	79 (88.8%)
Number of prophylactics used	2.5 ± 1.8	2.2 ± 1.8
Anxiety, % present [HADS‐A ≥ 8]	31 (34.4%)	24 (27.0%)
Anxiety, mean HADS‐A score	6.4 ± 4.0	6.0 ± 3.7
Depression, % present [HADS‐D ≥ 8]	35 (38.9%)	31 (34.8%)
Depression, mean HADS‐D score	6.5 ± 4.4	6.3 ± 3.9

Note

Values are absolute numbers with corresponding percentage, or mean ± SD.

Abbreviations: HADS‐A/HADS‐D, Hospital Anxiety and Depression Scale‐Anxiety/Depression; HIT‐6, Headache Impact Test‐6.

^a^ Maximal intervention, *n* = 85; minimal intervention, *n* = 89.

^b^ Simple analgesics: paracetamol, nonsteroidal anti‐inflammatory drugs.

^c^ Combined medication: combination of triptan and simple analgesics or combination drugs such as paracetamol and caffeine.

^d^ Commonly used prophylaxis for migraine.

^e^ History of use: current or past use of at least one type of prophylaxis.

**FIGURE 3:**
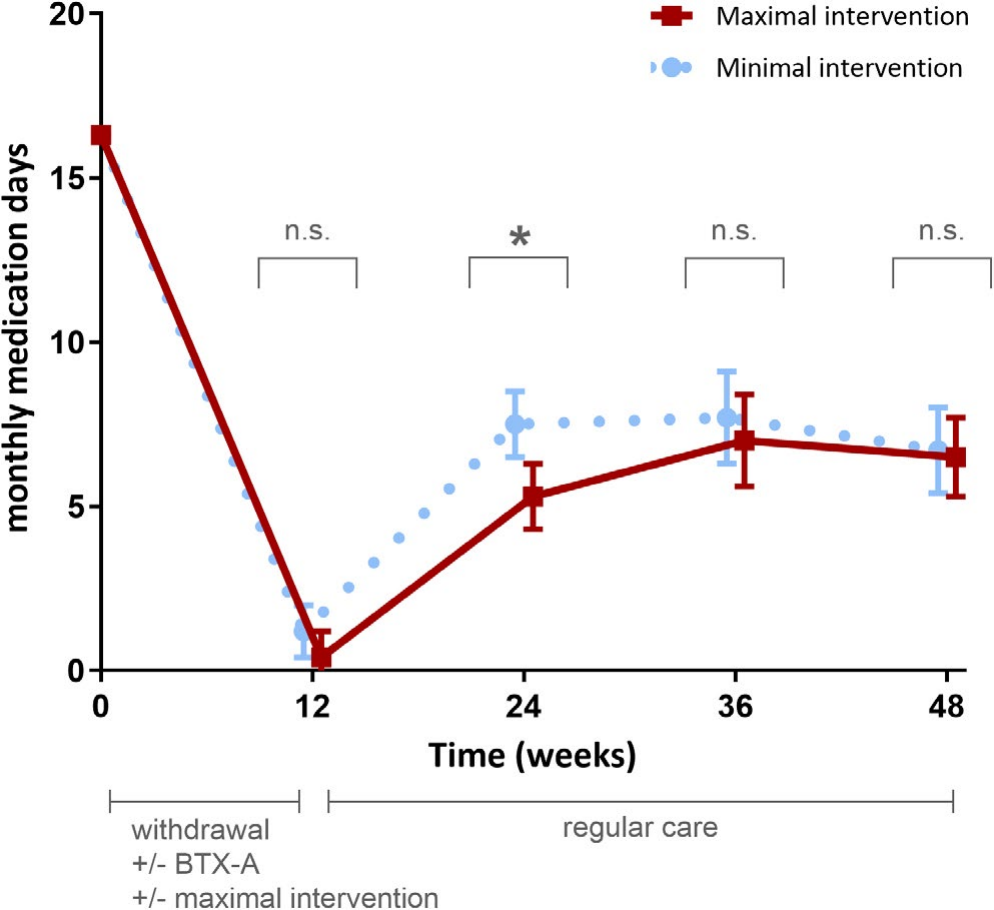
Effect of maximal versus minimal behavioural intervention (for the first period of 12 weeks) on acute medication use during withdrawal and after the withdrawal period. Depicted are adjusted means with standard errors derived from the linear mixed model analysis. Monthly medication days = monthly days with use of acute headache medication. BTX‐A, botulinum toxin A; n.s., nonsignificant; *p < 0.01. In the first 12 weeks, all patients had to withdraw from acute headache medication and were double‐blindedly randomized for BTX‐A and placebo. A concealed double‐blinded randomization was performed for maximal and minimal behavioural intervention in this 12‐week period. After these 12 weeks, patients who continued to have chronic migraine were offered open‐label BTX‐A; otherwise, usual care was provided by the treating physician without further behavioural intervention by the headache nurse. A detailed explanation of maximal and minimal intervention is in the Methods section [Colour figure can be viewed at wileyonlinelibrary.com]

Successful withdrawal, defined as ≤2 days/month escape use of acute headache medication in the first 12 weeks of the study, was achieved in 82 (93%) patients in the maximal intervention group, and 75 (86%) of those who received minimal intervention. The odds ratio for success was 2.44 (95% confidence interval [CI] = 0.83–7.23, *p* = 0.107) for maximal versus minimal intervention. The days with use of acute headache medication in this period was low in both groups, with no difference between groups (maximal intervention 0.39 vs. minimal intervention 1.15, mean difference = −0.76, 95% CI = −0.22 to 1.74, *p* = 0.128; Figure 3).

Patients in the maximal intervention group did have fewer monthly days with use of acute headache medication after 24 weeks (5.26 vs. 7.49, mean difference = −2.23, 95% CI = −3.76 to −0.70, *p* = 0.005). The difference between the two groups disappeared over time (mean differences after 36 weeks: −0.77, 95% CI = −2.66 to 1.12, *p* = 0.423 and after 48 weeks: −0.20, 95% CI = −1.90 to 1.49, *p* = 0.812; Figure 3).

The monthly migraine days decreased in both groups after withdrawal therapy, but did not differ between the maximal and minimal intervention groups (baseline: 15.32 vs. 14.90; Week 12: 8.57 vs. 8.68; Week 24: 9.44 vs. 9.91; Week 36: 10.71 vs. 9.08; Week 48: 9.80 vs. 8.64).

## DISCUSSION

This double‐blind randomized controlled trial suggests modest benefit of behavioural intervention for withdrawal therapy in MOH with reduced use of headache medication in the period after acute withdrawal. The odds of achieving successful withdrawal were numerically higher for the intensive behavioural intervention, albeit nonsignificantly. The medication use was both numerically lower and statistically different after withdrawal (Week 24). As the behavioural intervention was only provided during the withdrawal period itself (first 12 weeks), this effect gradually diminished during the long‐term follow‐up period of almost 1 year.

Hitherto, evidence for nonpharmacological interventions in the treatment of MOH has been mainly based on observational studies [15, 16, 25]. For headache disorders in general, contradictory conclusions have been drawn on existing data. A recent Cochrane review indicated a lack of good‐quality research on the efficacy of psychological interventions in migraine. A potentially higher response rate was suggested (risk rate for response = 2.21, 95% CI = 1.63–2.98), but based on trials with a high risk of bias [26]. Meta‐analyses using broader inclusion criteria, for instance, a population with both migraine and tension‐type headache, suggest efficacy of psychological treatment [3, 27]. Nevertheless, common ground in these studies is urgently needed for high‐quality clinical trials, minimizing risk of bias.

Our study has the major advantage that it conceals the behavioural intervention within another trial, which is a new and unique design in headache trials and provided an adequately blinded control group. Unblinding might occur by social interaction between trial participants on the dedicated research days. Due to the intensive versus minimal behavioural intervention principle, the two treatment arms were unlikely to be revealed by incidental contact between patients, especially as patients were not aware of the existence of this part of the study. Therefore, blinding was guaranteed, reducing bias by psychological mechanisms such as expectations and classical conditioning [4]. The implementation of such a design has to be performed with explicit approval of an ethical committee, as usually full informed consent from the patient is a key element for good clinical research practise. The current trial was considered within ethical boundaries, as patients provided informed consent for a clinical trial and were not harmed or aggrieved by the additional intervention study, as even minimal behavioural support was more than what is provided in most general neurology practices without a headache nurse.

Nonetheless, the concurrent drug trial might have influenced the results. The rates of successful withdrawal in our study are relatively high in both our groups (86% and 93%), as previous studies showed rates of 62%–85% [15, 16, 23, 28]. As patients would only receive subsequent open‐label BTX‐A in case of successful withdrawal, the drug trial may have contributed to the high withdrawal success rates in both groups during the first 12 weeks, constricting the differences between groups. A direct effect of the open‐label BTX‐A was highly unlikely, as open‐label BTX‐A did not influence the number of medication days [19], and patients with open‐label treatment were equally divided between both behavioural intervention groups. Also, the necessary contacts within the trial by research physicians or assistants, in which patients might have been motivated to some extent, or the effect of administration of a drug, whether verum or placebo, might have influenced the endurance of withdrawal, and could diminish the difference between interventions. This likely explains that the difference between the groups was not statistically significant after 12 weeks, although the magnitude of the odds ratio and the 95% CI indicate effect. Furthermore, a significant problem with MOH is relapse into overuse of acute medication. Therefore, we aimed to restrict patients on acute medication after the acute withdrawal period. Our study suggests benefit of behavioural intervention with reduced use of headache medication in the period after the acute withdrawal period. As the behavioural intervention was only provided during the withdrawal period itself (first 12 weeks), this effect gradually diminished during the long‐term follow‐up period of almost 1 year, which we also expected, as only limited care was provided by the treating physician, with a visit to our headache clinic only once per 3 months.

During the behavioural intervention, the consults not only were used to educate on medication overuse and to increase intrinsic motivation to initiate medication withdrawal using motivational interviewing techniques, but also aimed to enhance acknowledgement and acceptance of the influence of migraine on the various aspects of life in general. Furthermore, alternative behavioural strategies to cope with the untreated pain, including relaxation, were discussed, and a value‐based approach was introduced to establish activities. By reviewing main values, the headache nurse helped to reorganize daily life in such a way that despite the limitations due to migraine or the withdrawal, the most meaningful activities could be regained. We hypothesize that these latter aspects of the consults induced the effect of the intervention beyond the withdrawal therapy itself, with significant lowering of use of headache medication after 24 weeks. As we stopped the behavioural intervention with the nurse after 12 weeks, this explains the diminishing effect during the long‐term follow‐up of 1 year. Underlying biological factors and comorbidities such as depressive symptoms and anxiety (i.e., factors that may influence relapse into chronification of migraine) might have played an important role in this diminishing effect as well [7, 10, 28]. In the first year after withdrawal therapy, high rates of relapse into medication overuse of up to 40% are reported [29], posing a challenge in maintaining the effect of withdrawal therapy. Prolonged intensified intervention by a headache nurse after withdrawal might reduce relapse rates [30, 31].

Our findings in this randomized and blinded trial affirm previous results on the benefit of multidisciplinary care during withdrawal in observational studies. Our previous retrospective study on behavioural intervention by a headache nurse showed an increased rate of successful withdrawal as well, but did not include a long‐term follow‐up [15]. A Danish observational study suggested that multidisciplinary approaches during withdrawal therapy are effective. In that study, a structured schedule with both group and individual therapy by a nurse, psychologist, and physiotherapist was compared and found not to be superior to a structured schedule with a headache nurse alone. Interpretation from this comparison has to be done with caution, however, because both groups also differed in withdrawal strategy [16]. Furthermore, the effect perceived by patients of an educational or behavioural intervention might also be on improvement of pain coping strategies, not only on medication intake. An open‐label randomized controlled trial suggests improvement of pain coping strategies upon an educational intervention, although between‐group differences with the control group were not established [18]. Outcome measures on pain coping were not included in this trial, and further research on this aspect is deemed necessary. In primary care, a cluster‐randomized controlled trial in 60 MOH patients among general practitioner (GP) practices showed effectiveness of a brief intervention. Feedback on their dependency behaviour resulted in reduced medication use [32]. This study suggests that behavioural interventions may be implemented in GP practices as well.

In general, there is an ongoing debate on the necessity of withdrawal, which is raised again regarding studies in chronic migraine patients with medication overuse and the effect of antibodies against calcitonin gene‐related peptide (CGRP) or its receptor [33, 34, 35, 36]. Nonetheless, cessation or lowering of the overused medication remains an important factor in the treatment of medication overuse headache, resulting in a significant reduction in headache days in the majority of patients [7, 8, 15, 16, 21, 22, 23, 37]. Various strategies are being used [11, 15, 16, 21, 22, 23, 28, 37] in which complete withdrawal seems more effective compared to lowering of the medication [23], and it is suggested that withdrawal can be advised in combination with preventatives [11]. However, in our CHARM study, withdrawal combined with BTX‐A did not afford any benefit over withdrawal alone [19]. This current substudy suggests that behavioural intervention may enhance the efficacy of withdrawal. Although a cost‐effectiveness analysis was not included in this study, the absolute costs of withdrawal and the time invested by the headache nurse are low, especially compared to more costly preventatives such as BTX‐A, which needs to be administered by a specialized nurse or physician, or monoclonal antibodies against CGRP or its receptor. Both the minimal behavioural intervention and the more intensive behavioural intervention resulted in a high percentage of successful withdrawal, but the effect in the intensive groups was more prolonged. Hence, we would advise a prolonged intervention with multiple telephonic contacts, which are relatively inexpensive. Furthermore, with the COVID pandemic, e‐consultations and e‐coaching have been rapidly developed in many countries, which are very cost‐effective.

In conclusion, our unique large concealed double‐blind randomized controlled trial study suggests benefit of implementation of a behavioural intervention for withdrawal therapy in MOH with reduced use of headache medication in the period after withdrawal. Future studies may aim at investigating long‐term behavioural intervention that can be provided by a trained nurse. The principles of a concealed study design with a low‐dose versus high‐dose principle can also be useful in the research field of behavioural interventions in other central nerve system disorders.

## CONFLICT OF INTEREST

G.M.T. reports consultancy support from Novartis, Allergan, Lilly, and Teva, and independent support from Dutch Organization for Scientific Research, the Dutch Heart & Brain Foundations, IRRF, and Dioraphte. The other authors report no relevant disclosures.

## AUTHOR CONTRIBUTIONS

**Judith A. Pijpers:** Conceptualization (equal), data curation (equal), formal analysis (lead), investigation (equal), methodology (equal), project administration (equal), software (equal), validation (equal), visualization (equal), writing–original draft (lead), writing–review & editing (lead). **Dennis A. Kies:** Conceptualization (equal), data curation (equal), investigation (equal), methodology (equal), project administration (equal), writing–review & editing (equal). **Erik W. van Zwet:** Data curation (equal), formal analysis (equal), methodology (equal), validation (equal), writing–review & editing (equal). **Frits R. Rosendaal:** Conceptualization (equal), data curation (equal), methodology (equal), writing–review & editing (equal). **Gisela M. Terwindt:** Conceptualization (equal), data curation (equal), funding acquisition (lead), investigation (equal), methodology (equal), resources (equal), supervision (lead), validation (equal), visualization (equal), writing–review & editing (lead).

## Data Availability

The data that support the findings of this study are available from the corresponding author upon reasonable request.
